# Targeting the Substrate: Mechanism-Based Ablation Strategies for Persistent Atrial Fibrillation

**DOI:** 10.3390/jcm14145147

**Published:** 2025-07-20

**Authors:** Gabriela-Elena Marascu, Alexandru Ioan Deaconu, Raluca-Elena Mitran, Laura Adina Stanciulescu, Radu Gabriel Vatasescu

**Affiliations:** 1 Faculty of Medicine, Carol Davila University of Medicine and Pharmacy, 050474 Bucharest, Romania; gabriela.marascu@yahoo.com (G.-E.M.); laura-adina.stanciulescu@drd.umfcd.ro (L.A.S.); radu_vatasescu@yahoo.com (R.G.V.); 2 Cardiology Department, Clinical Emergency Hospital, 014461 Bucharest, Romania; ralucamitran972@gmail.com

**Keywords:** atrial fibrillation, pulmonary vein isolation, radiofrequency, ablation, personalized approach

## Abstract

Pulmonary vein isolation (PVI) is the cornerstone of atrial fibrillation (AF) ablation, especially effective in patients with paroxysmal AF, where the pulmonary veins (PVs) are the primary triggers. More complex arrhythmogenic mechanisms are involved in persistent AF (PsAF), and PVI alone may not be sufficient. Personalized, substrate-based ablation strategies are increasingly used and can significantly enhance outcomes in PsAF patients. While radiofrequency ablation remains the gold standard, cryoablation provides effective PVI, and pulsed field ablation is emerging as a safer, promising alternative. Advanced mapping techniques may better target scar areas responsible for arrhythmogenesis, optimizing procedural results. While still in development, artificial intelligence and machine learning enable more personalized and precise ablation strategies and may improve long-term outcomes.

## 1. Introduction

The mechanisms behind atrial fibrillation (AF) are complex and involve multiple factors. AF typically begins with triggers activating vulnerable areas, with arrhythmias being more sustained as the substrate progresses [1]. The pulmonary veins (PVs) are crucial in the development of AF, and their isolation has high success rates for paroxysmal AF (PAF). However, persistent AF (PsAF) ablation is less effective due to challenges in identifying its underlying mechanisms outside PVs, which leads to AF perpetuation [2]. Numerous theories have been proposed to clarify the persistence of this type of AF, varying from a singular localized focal and reentrant origin to widespread biatrial multiple wavelets [2]. This reentrant activity can be intramural, endocardial, or epicardial, which limits their accessibility for mapping and ablation procedures [2].

Recent insights into the mechanisms that promote trigger activation and AF persistence, along with advancements in mapping technology, suggest that therapeutic strategies should focus on eliminating triggers and modifying the atrial substrate [1]. Novel imaging techniques can provide data on atrial tissue properties, helping to stratify patients at higher risk for AF and to tailor ablation strategies. This includes non-invasive methods like detecting atrial strain via echocardiography, left atrial (LA) wall thickness, and atrial fibrosis through cardiac magnetic resonance (CMR) [1,3].

Circumferential pulmonary vein isolation (PVI) is the cornerstone of treatment for AF, regardless of its type or duration. No ablation technique has shown consistent superiority over this method in preventing long-term atrial arrhythmia recurrences [2]. PV reconnections are the main cause of recurrence in PAF, while the mechanisms for recurrent atrial arrhythmias when PVs are persistently isolated after catheter ablation of PsAF are not well defined. The occurrence of PV reconnections in recurrent AF may be less frequent now that ablation technology has advanced to include contact force and irrigated catheters to create long-lasting ablation lesions [4]. Further research is needed to identify the mechanisms of AF that can improve outcomes for patients undergoing ablation for PsAF.

## 2. What Are the Basic Mechanisms of AF?

AF is commonly triggered by focal sources, particularly from PVs. It is maintained by multiple small reentrant wavelets within the atrial myocardium. Atrial fibrosis and, more recently, LA wall fat infiltration play a significant role in creating conduction variability, enhancing favorable conditions for reentry [5,6]. The goal of standardized empiric ablation techniques, such as PVI and linear lesion sets, is to eliminate both the initial triggers and the structural pathways that sustain reentrant circuits, thereby preventing AF recurrence.

The mechanisms of AF are complex and not fully understood, involving interactions among triggers, substrates, and modulators such as ionic changes, genetic factors, and neuro-humoral influences [2]. Frequent rapid discharges from PVs may cause AF due to enhanced automaticity or triggered activity [7]. Reentry is not typically maintained in a normal atrium; however, intermittent AF episodes result in electrical and structural remodeling, with fibrosis, fat infiltration, and chamber dilatation, which promote reentry through conduction abnormalities [5,6,7].

The pathophysiological mechanisms involved are intricate, and there is a growing recognition that various comorbidities may play a role in promoting atrial remodeling associated with AF. Additionally, recent studies have emphasized that the risk of AF varies over time and that fluctuations in accompanying conditions add to the complexity of AF dynamics [8]. AF is often a chronic condition that begins with intermittent, short-term episodes that return spontaneously to normal sinus rhythm [8]. Factors linked to the transition from PAF to PsAF include advancing age, left atrial enlargement, valvular heart disease, and conditions like hypertension, diabetes, obesity, heart failure, coronary artery disease, chronic kidney disease, chronic obstructive pulmonary disease, previous transient ischemic attack or stroke, and obstructive sleep apnea [7]. More recently, even the unnecessarily extended LA ablation during PVI for PAF was also associated with the risk of progression to PsAF [9]. Risk factor management and lifestyle changes could aid in both the prevention and treatment of AF.

Major pathologic processes that contribute to AF development include ectopic activity and reentry [8,10]. Ectopic activity may result from abnormal automaticity and early or delayed afterdepolarizations (EADs and DADs) [8]. EADs occur due to the prolonged repolarization duration, allowing L-type calcium channels to recover from their initial inactivation and generate a secondary depolarization during the action potential (AP). DADs are linked to spontaneous calcium release from the sarcoplasmic reticulum via the type-2 ryanodine receptor during diastole, leading to an inward depolarizing current generated by the sodium–calcium exchanger type 1 [8].

The triggered activity can result in a unidirectional block and initiate reentry. Reentry is considered the predominant AF-maintaining mechanism and is favored by a short and/or variable effective refractory period (ERP) and slow, heterogeneous conduction [8]. Electrical remodeling is primarily a result of AF, even though it might exacerbate the condition due to changes in cardiac ion channels. For patients with PAF who were in sinus rhythm, repolarization duration remains unaltered; for individuals with (long-standing) chronic AF, the reduction in ERP is reversible following cardioversion [8]. Additionally, reentry-promoting slow heterogeneous conduction might result from the loss of electrical cell-to-cell connection via gap junctions [8]. The autonomic nervous system, renin–angiotensin–aldosterone system, inflammation, and hemodynamic alterations are significant mediators of elevated AF risk induced by risk factors and comorbidities [8].

Genetic predisposition contributes to early-onset familial cases of AF, with up to one-third of patients carrying genetic variants that increase AF risk [2]. Genome-wide association studies have identified at least 30 gene loci linked to AF [2,11,12,13,14,15,16,17,18]. These variants likely create a background vulnerability rather than directly causing AF. The development of additional risk factors over time, such as aging or cardiac remodeling, can trigger AF [10].

**Figure 1:**
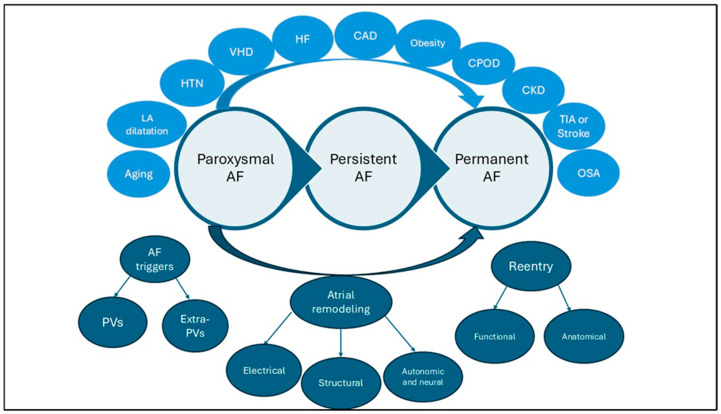
Atrial fibrillation progression. A simplified scheme that describes atrial fibrillation evolution over time, including risk factors (upper part) and mechanisms (below part). AF: atrial fibrillation; PVs: pulmonary veins; LA: left atrium; HTN: hypertension; VHD: valvular heart disease; HF: heart failure; CAD: coronary artery disease; CPOD: chronic pulmonary obstructive disease; CKD: chronic kidney disease; TIA: transient ischemic attack; OSA: obstructive sleep apnea.

The mechanisms underlying AF are classically described as mechanisms responsible for its initiation (triggers) and mechanisms responsible for its perpetuation and progression toward longer-lasting AF forms [2,10] (Figure 1).

**AF Triggers**

The role of PVs in triggering AF was confirmed in several studies. Compared to the atrial cells, PV cells have a higher resting membrane potential, a lower amplitude of the AP with a shorter duration, and a smaller maximum phase 0 upstroke velocity [2]. Transient outward potassium current and L-type calcium current are lower in PVs, but slow and rapid delayed rectifier currents are higher [2]. Atrial myocytes at the entrance of PVs have abrupt changes in their fiber orientation, leading to slow conduction and reentrant activity at the junction between PVs and LA [2]. Additional ectopic sources initiating AF were identified outside PVs, and can be located in the vena cavae, crista terminalis, coronary sinus, posterior wall (PW) of the LA, ligament of Marshall, interatrial septum, and appendages [1,2].

Non-PV triggers are more prevalent in PsAF and were predicted by female gender, older age, severe left atrial fibrosis, obesity, sleep apnea, reduced left ejection fraction, mechanical mitral valve, hypertrophic cardiomyopathy, and late recurrence after persistent PVs antral isolation [2,19]. Meng et al. recently suggested that PVs act more as echo chambers, amplifying fibrillatory wave front activity instead of serving as primary AF drivers. This offers a new understanding of why PVI is crucial in treating PsAF and suggests that similar mechanisms in areas of abnormal substrate within the atria may contribute to maintaining PsAF [20].

Strong risk factors for AF include obesity, which is linked to both incident and persistent AF. Research indicates that to reduce the burden of AF in patients who are overweight or obese, at least a 10% weight loss should be the goal [21].

Fat infiltration in the subepicardium has been implicated in conditions like Brugada syndrome and early repolarization syndrome. Some researchers propose a subepicardial cardiomyopathy as a distinct disease entity. Epicardial Adipose Tissue (EAT) directly leads to arrhythmias via structural barriers, electrotonic coupling between adipocytes and myocytes, and secretome signaling (microRNAs, cytokines, and extracellular vesicles) [22].

Increased EAT volume correlates with atrial AF and ventricular tachycardia [22]. Studies indicate that reducing EAT volume through weight loss via diet, exercise, or medications like GLP-1 receptor agonists and SGLT2 inhibitors may help prevent arrhythmias. Future therapies might involve capturing EAT secretome signals or modifying myocardial cells to resist EAT-induced effects [22]. The remodeling of EAT is particularly associated with persistent forms compared to paroxysmal ones. The likelihood of arrhythmic recurrence following ablation appeared to be linked to more EAT [23,24].

Huber et al. showed that greater LA EAT dispersion observed in pre-procedural cardiac CT scans was linked to an increased risk of AF recurrence within one year after PVI [23]. A randomized trial with 124 obese PsAF patients found that LA EAT creates an arrhythmogenic substrate and increases the risk of driver development. In these patients, driver ablation improved AF termination rates and long-term outcomes [25]. A meta-analysis of 10 observational retrospective studies involving 1840 AF patients found that EAT is associated with a higher risk of AF recurrence after catheter ablation. This association was notably more common among Asian patients, those under 60 years old, and during long-term follow-up (over one year) [26].

However, not only EAT is involved in PsAF, but more recently, fat infiltration of LA walls was described; it seems to affect a significantly higher degree of patients with PsAF compared to PAF [5] and also appears to be involved in PV reconnections after radiofrequency ablation [6].

In an observational study, Landra et al. found that intramyocardial fat (inFAT) identified by pre-procedural multidetector computed tomography (MDCT) overlapped with about two-thirds of the reconnected sites in PVs following ablation. Segments with reconnections showed higher inFAT volumes, suggesting that inFAT may play a role in late PV-LA reconnections after PVI [6].

Saglietto et al. found that fatty infiltration of the LA is independent of the body mass index and increases with the severity of AF, from minimal levels in controls to high levels in patients with PsAF, suggesting an arrhythmic phenotype gradient [5]. Even without marked LA enlargement, patients may develop de novo PsAF if significant fatty infiltration is present in the LA [5]. These insights highlight that imaging-based assessment of atrial fat, especially via MDCT or CMR, could help identify high-risk patients and guide more personalized AF management strategies, particularly in those lacking conventional structural changes.

**Perpetuation of AF**

Structural and electrophysiological atrial changes lead to AF perpetuation [1]. Reentry constitutes an important mechanism in the maintenance of AF [1].

- Functional reentry: Reentrant activity in the absence of underlying substrate or anatomical obstacles.
  ○ The leading circle concept: Reentry happens in a circuit that matches the wavelength in size, featuring a central core that remains continuously refractory or unexcited [10]. Centripetal waves approaching the center sustain this subsequent refractoriness. When there is a slow conduction velocity or a short refractory period, it results in a reduced wavelength, making the spontaneous cessation of AF unlikely [2].
  ○ Spiral wave reentry (rotor concept, self-sustaining rotational systems with a spiral wave structure [1]): A specific area of reentry where the curved wavefront and the wave tail intersect at a singular point, and where the central tissue is not in a refractory state [10].

Focal ectopic discharges are necessary for the onset of AF in a susceptible substrate. Furthermore, these discharges can sustain AF when they happen frequently at a high rate. The predominant mechanisms responsible for the continuation of long-standing AF episodes in most patients involve multiple reentrant circuits or one or more rotors with fibrillatory conduction [10]. Vijayakumar et al. demonstrated that macro-reentry wave fronts, rotors, and both single and repetitive focal sources in the left and right atria maintain PsAF. These factors lead to multiple wavefronts propagating simultaneously throughout the biatrial structure. The wavefronts frequently break and collide, with these breaks likely resulting from structural remodeling of the atria associated with PsAF [27].

- Anatomical reentry: Reentry happens when there is a unidirectional block and slow conduction, resulting in a shorter wavelength than the circuit’s length. Such conditions are often found in the atria of AF patients, especially when fibrosis is present [2]. AF drivers are more commonly detected in the LA, whereas drivers in the right atrium were observed in 20% of the cases [2,28]. Simultaneous biatrial septal mapping in patients with PsAF shows that the left and right sides of the interatrial septum act as electrically separate structures, promoting reentrant activity through conduction slowing and block, further supporting the persistence of reentrant wavefronts [29].
- Atrial remodeling comprises both structural and functional changes that contribute to atrial arrhythmias, such as electrical, structural, and autonomic remodeling [10].
  ○ Electrical remodeling: AF and rapid arrhythmias affect ion channel expression and function. The high atrial rate during AF triggers protective mechanisms that limit calcium entry by inactivating calcium currents, which shorten the refractory period, and enhance potassium currents, which increase repolarization. This shortens the AP duration, increasing atrial vulnerability and sustaining AF. Additionally, impaired calcium handling contributes to contractile dysfunction and ectopic activity [1,10]. Electrical remodeling can contribute to early recurrence after cardioversion, progression from paroxysmal to more persistent forms, or the development of drug resistance [1]. Additionally, it is responsible for modifying the expression and localization of connexins, leading to conduction abnormalities [1].
  ○ Structural remodeling is defined by alterations in cellular ultrastructure and tissue characteristics that result in atrial dilatation and fibrosis [1]. Fibrosis represents the most important structural change that induces AF [1,10]. It can be reactive (located at the interstitial space) or reparative (replaces dead myocytes) [10]. Fibrosis increases the separation of myocytes in subendocardial atrial bundles and between the endocardial and epicardial layers, leading to endo-epicardial dissociation, which creates barriers to the propagation of activation wavefronts [2]. Beyond fibrosis, the atrial structure undergoes changes such as fatty infiltration, the presence of inflammatory cells, tissue necrosis, and amyloid accumulation. Adipose tissue exerts a paracrine effect by releasing adipokines that possess profibrotic characteristics. Additionally, it creates obstacles to wavefront conduction and promotes reentrant circuits [2,7].

Patients with PsAF have a greater extent of atrial fibrosis and LA wall fat infiltration compared to those with PAF. Furthermore, the rate of AF recurrence following PVI was associated with the level of LA fibrosis observed through high-resolution CMR [30]. Additional research established the link between decreased bipolar voltage during AF and sinus rhythm and delayed enhanced atrial areas on CMR imaging [30]. The DECAAF II randomized trial showed that baseline fibrosis was a predictor of outcomes in AF ablation, particularly at higher levels of fibrosis; however, there were no statistically significant differences noted in the time to first recurrence of arrhythmia [31].

 ○ Autonomic and neural remodeling: Neural remodeling includes an increase in atrial innervation. Gould et al. demonstrated an increased atrial sympathetic innervation in patients with PsAF [2,32]. Atrial sympathetic innervation was reported to increase in response to rapid rates of AF. Patients with heart failure have been found to have enlarged cardiac ganglia and more sympathetic/parasympathetic fibers in their PVs and posterior left atrium, promoting AF maintenance [1].

It is important to note that structural remodeling and fibrosis, as opposed to electrical remodeling, are likely irreversible and lead to the transition from paroxysmal to persistent, long-standing, or permanent forms [2]. Both AF-related remodeling and remodeling brought on by aging, cardiac disease, or comorbidities contribute to the progression of AF, and early therapies are crucial to preventing disease progression [2,10].

But not every patient experiences each step, and the period spent in each phase can differ significantly, including those whose initial AF presentation is a persistent/permanent form, or those who have PAF that will never progress [33]. Patients with PsAF phenotype from the beginning are younger and have higher heart failure rates and lower left ventricular ejection fraction than those whose initial PAF progressed to PsAF [34], possibly related to higher arrhythmia burden. They are usually taller men and respond similarly with PAF patients to PVI (unpublished observations), suggesting an anatomic predisposition to PsAF (larger absolute LA volume, but normal when indexed to height). By comparison with PAF, this PsAF phenotype exhibits similar atrial remodeling and comparable ablation success, with no significant differences in time to AF recurrence or AF burden after one year [34].

## 3. Mapping Technologies for Persistent Atrial Fibrillation

Understanding the mechanisms of AF is crucial for improving ablation methods, and mapping plays an essential role. Nevertheless, mapping technologies are exposed to spatiotemporal instability, resulting in poor reproducibility. Indeed, reports of ablation using different mapping methodologies have shown contradictory results.

Previously, mapping systems mainly provided anatomical maps for lesion placement. Developments in this technology now allow for the activation and mapping of the wavefront across the entire atrium during AF [35]. Research suggests that AF may be sustained by organized high-frequency regions called “drivers”, consisting of multiple foci and rotational wavefronts. Recent mapping advancements for PsAF ablation employ systems that can map the entire chamber during AF and visualize these drivers or help categorize specific electrograms, like complex fractionated electrograms [35].

The FIRM approach (focal impulse and rotor modulation) is an invasive mapping method that identifies electrical rotors and focal impulses. Recent multicenter trials and systematic reviews have shown significant variability in success rates for AF rotor and driver ablation, with outcomes not exceeding those of conventional PVI [1]. In order to eliminate rotors and focal sources, prospective studies assessed the substrate modification guided by repetitive and/or organized similar electrography. According to the findings of these investigations, areas in the atria with uniform activation waveforms may be found using electrogram similarity analysis, independent of spatial wavefront propagations [36,37]. The PRISM-guided ablation was shown to reduce the recurrences in PsAF patients [38,39].

A 3D visualization of the anatomy of any heart chamber is possible with electroanatomical mapping (EAM) systems, which can be made with point-by-point acquisition using an ablation catheter or, more commonly, with specialized multi-electrode catheters. Real-time 3D EAM maps can be integrated with pre-acquired cardiac computed tomography or CMR images. Both CARTO and Ensite offer optional algorithms for the automated identification of complex fractionated atrial electrograms (CFAEs) during AF, along with the capability to mark corresponding regions on the 3D anatomical map [7].

Using CARTOFINDER, high-density panoramic imaging can help adjust wide-area PV ablation by detecting rotational and focal activity. The CARTOFINDER-guided ablation may be more effective than traditional PVI and removal of extra-PV triggers in PsAF patients [40]. On the other hand, Takahashi et al. showed that CARTOFINDER mapping-guided ablation of focal activation areas as an adjuvant to PVI did not decrease the arrhythmia recurrence rate in patients with PsAF when compared to PVI alone [41].

Mapping-guided ablation may fail to improve clinical outcomes because endocardial mapping does not detect all AF drivers. The endocardium and epicardium show different atrial propagations, and unmapped areas can miss triggers. Even with both surfaces mapped, intramural reentry may remain undetectable and contribute to AF [41].

Endocardial–epicardial dissociation (EED), discordant wavefronts (DWFs), and epicardial breakthrough are phenomena seen in AF, resulting from complex 3D electrical and structural interactions in the atria [42]. Electrophysiological differences—such as refractory periods and conduction velocities—between the epicardial and endocardial layers, in addition to their anatomical characteristics, may contribute to differences in conduction patterns and electrical dissociation during AF [42]. High-density simultaneous mapping in goat atria revealed that as the complexity of AF increases, AF induced by rapid atrial pacing exhibits the highest extent of EED. Additionally, both the occurrence of epicardial and endocardial breakthroughs increases along with the progression of AF to a persistent form [42,43]. In another study, simultaneous endocardial–epicardial mapping in patients with PsAF detected ~50% functional EED with temporal heterogeneity [44].

In order to detect the substrate of AF, a novel method called electrographic flow mapping can produce spatiotemporally reproducible maps and visualize the real-time atrial electrical wavefront propagation. Electrographic flow can estimate cardiac action potential flow across the atrial myocardium based on transmembrane voltage variations indicated by unipolar electrograms, and make the difference between active and passive sources, without the need for phase analysis or other conventional signal processing techniques [45].

Functional mapping using a short-coupled atrial extrastimulus approach in sinus rhythm was demonstrated by Silva Garcia et al. to uncover hidden slow conduction zones, which are more frequently identified in PsAF [46]. The researchers also found that areas of CFAE detected during AF did not always co-localize with hidden slow conduction zones.

Since electrophysiological examinations are often catheter-based, the anesthesia needed for these procedures may affect and suppress the results. Scarring from the ablative process that follows can influence future mapping. Such changes are not a part of non-invasive body surface mapping (ECGI, or electrocardiographic imaging), which is the only modality capable of simultaneous biatrial electrical characterization [7,47]. In PsAF patients, unstable reentry circuits with dynamic spatiotemporal activity were identified as the main sustaining mechanism using this mapping system [7]. ECGI maps may incorrectly identify rotational drivers, similar to findings in the TARGET-AF1 study, where more rotating drivers failed to respond to ablation compared to focal drivers [48,49]. This suggests either that rotational drivers are often misidentified or that focal drivers play a more crucial role in sustaining AF. The authors recommend close examination of rotational drivers while prioritizing the ablation of focal drivers [49]. In the TARGET-AF2 trial, most drivers were rotational, mainly located in the PW of the LA. There was no significant difference in ablation response between rotational and focal drivers [50].

Compared to voltage-based mapping, non-contact charge density mapping (CDM) has been shown to offer superior resolution for localized electrical activation. The non-contact CDM may reveal potential ablation targets across the LA substrate, emphasizing the importance of extending ablation beyond PVs and posterior wall isolation (PWI), especially when addressing abnormal conduction patterns [51]. An individualized ablation strategy that targets the conduction pattern core (CPC) via core-to-boundary ablation guided by CDM can be a successful approach for treating PsAF, with a positive 24-month outcome, according to a prior observational study [52]. The number of CPCs was also shown to be associated with the length of PsAF, and following PVI, there was no significant change in the spatiotemporal stability and incidence of CPCs [52].

Advanced computational analysis of atrial electrograms (EGMs) aiming to quantify subtle features like dispersion, entropy, and conduction heterogeneity that correlate with the AF substrate. Spatiotemporal dispersion indicates variability in EGM morphology and timing, reflecting disorganized conduction.

Electrogram entropy measures the unpredictability or complexity of an EGM. Shi et al. introduced Multiscale Entropy (MSE) mapping to identify high-complexity regions in PsAF [53]. In their prospective study of 108 patients, the highest-entropy regions were targeted after PVI, with ablation of 1–3 high-MSE areas leading to AF termination in ~35% of cases. Notably, higher left-atrial entropy predicted better outcomes, indicating that uneven EGM complexity is a marker of arrhythmogenic substrate [53]. Electrogram morphology recurrence (EMR) is an innovative metric that measures how frequently an EGM waveform repeats at a specific site. It utilizes recurrence percentage (Rec%) and the cycle length of the most recurrent morphology (CL_R_) derived from high-density mapping data. Yoo et al. demonstrated that EMR can identify stable reentrant drivers, finding that high Rec% and short CL_R_ are linked to sustained rotational circuits and areas with high parasympathetic innervation [54]. Vector Field Heterogeneity (VFH) helps quantify irregular conduction patterns by measuring abrupt changes in conduction vectors from high-density recordings. VFH may outperform traditional measures in detecting disorganized propagation and may identify critical areas sustaining AF [55]. Increased conduction heterogeneity correlates with more advanced AF stages, often coinciding with fibrosis [56].

A study of 27 AF patients found that a novel metric, left atrial spatial entropy (LASE), was significantly lower in PsAF compared to PAF and in fibrotic atrial substrate versus normal tissue [57]. LASE, using Shannon entropy to quantify voltage distribution, distinguished abnormal substrate with an AUC of ~0.81 (optimal cutoff ≈6.06, achieving 80% sensitivity and specificity) [57]. Patients with the lowest entropy values were more likely to experience AF recurrence on follow-up, indicating that entropy measures of EGM heterogeneity can effectively characterize atrial substrate and correlate with AF type and outcomes, independent of the rhythm during mapping [57].

## 4. Standard or Personalized Approach for Radiofrequency Ablation?

Radiofrequency ablation (RFA) is a widely used treatment for AF, and its success largely depends on selecting the appropriate ablation strategy. PVs are important trigger sites of PAF, and their electrical isolation represents the conventional strategy, with a high rate of freedom from arrhythmia in patients without comorbidities.

Electrical reconnection of previously isolated PVs is the main cause of AF recurrences, regardless of the energy source. PVI durability varies widely (3% to 53%) after thermal ablation, often due to factors like inadequate temperatures, poor balloon-to-vein fit, low contact force, and tissue impedance changes [58]. On the other hand, insufficient radiofrequency delivery in the thickest myocardial areas can lead to non-continuous or non-transmural lesions [3]. Using a PVI-only strategy with ablation index adjusted based on local LA wall thickness resulted in high recurrence-free survival after a median follow-up of 16 months [3].

Wasmer et al. conducted a retrospective study that enrolled 149 patients with AF (80 with PAF and 69 with PsAF), and found that most with AF recurrences after PVI had at least one reconnected vein, regardless of AF type. AF progression occurred in both directions, independent of the initial AF classification. On the other hand, they reported that about 13% of patients with PsAF improved to PAF after their first PVI. They concluded that using circumferential PVI as the first approach for all AF types and reserving more extensive ablation for those with recurrence despite durable PVI [59]. William et al. randomized patients with PsAF that underwent RFA to PVI and PWI or PVI alone, and showed that in those undergoing redo ablation, PV reconnection was observed in 54.5% (mean number of reconnected PVs 2.2 ± 0.9) and PW reconnection in 75% [60].

The Ablation Index Registry study included 490 patients with PAF (predominantly) and PsAF concluded that at the redo procedure, the likelihood of finding all four veins still isolated was 30%, and no clear predictors were identified for either acute or delayed PV reconnection [61].

Another prospective study enrolled 30 patients with PsAF who underwent a planned remapping at 6 months after the index procedure, irrespective of AF recurrences (the CLOSE-guided approach), and showed that PV reconnections were present in 24% of PVs and durable isolation of all PVs in 58% of patients. Among patients who underwent repeated PVI due to PV reconnections, as well as those who received additional ablation for recurrence despite having durable PVI, 35% experienced arrhythmia recurrence 12 months after the redo procedure [62].

A recent meta-analysis that included over 7000 patients with AF reported that the PVI durability per patient and per vein was similar between thermal ablation and pulsed field ablation (PFA), with no differences in the reconnection rates of individual PVs. On the other hand, this study found that PV reconnection occurred significantly more often after RFA compared to PFA [58]. Kueffer et al. showed that in patients with PsAF who underwent PVI with RFA, cryoablation, or PFA, the PVI durability and the individual veins’ durability were not different regardless of the index ablation technology. Also, they found that PFA and CBA were associated with higher arrhythmia-free survival compared to RFA [63].

In PsAF, however, additional arrhythmogenic atrial sites are responsible for AF maintenance, and PVI alone may not be sufficient [30]. In these patients, a personalized strategy is often necessary, involving additional techniques such as linear ablation, ethanol infusion into the vein of Marshall, low voltage area (LVA) ablation, extra-PV trigger ablation, complex fractionated electrograms ablation, rotor ablation, ganglionated plexi ablation, and targeting atrial tachycardias that develop post-ablation. Despite adjustments by adding linear ablation and defragmentation, the success of catheter ablation in PsAF has remained substantially lower, with frequent recurrence of atrial arrhythmias [27]. A meta-analysis conducted by Saglietto et al. showed that a comprehensive approach combining PVI, LA linear lesions, and targeting of extra-PV sources, despite varying definitions across studies, was linked to a lower risk of recurrent atrial tachyarrhythmias compared to PVI alone [64].

Prospective randomized trials have not shown an incremental advantage from linear ablation beyond PVI. Using RFA to create linear lesions may raise the risk of left atrial tachycardias [65]. According to the PROMPT-AF trial, ethanol infusion of the vein of Marshall and PVI, along with a linear ablation strategy using radiofrequency energy that included the mitral isthmus, LA roof, and cavotricuspid isthmus, significantly increased the patients’ freedom from atrial arrhythmias within 12 months when compared to PVI alone [65]. In a recent prospective randomized trial, the Marshall plan, which includes PVI combined with additional ablation, including the vein of Marshall ethanol infusion and three lines of block at the mitral, dome, and cavotricuspid isthmuses during the first procedure, significantly improved freedom from any atrial arrhythmia at 12 months when compared to PVI alone [66].

In the AFACART study, 118 patients with PsAF lasting less than a year had their ablation guided by non-invasive mapping. Ablation focused on the drivers the system had identified, and when AF could not be stopped, linear ablation and PVI were used. In 64% of the cases, AF was ended by ablation directed at the drivers’ sites. With additional PVI and atrial linear ablation, the AF termination rate increased to 72%. Extra-PV sources were essential to PsAF, and ablation of these sources usually ends AF with rhythm control of up to 77% after 1 year. However, atrial tachycardia recurrence remains significant, indicating that a more selective approach targeting fewer drivers may be necessary [67].

The benefits of extensive linear ablation in PsAF patients were noted in a multicenter randomized study. A total of 214 patients were randomly assigned to Group I (PVI + LA roof line+ LA anterior wall line) and Group II (PVI + LA roof line), with or without mitral isthmus linear ablation. Patients in Group II achieved a higher rate of successful termination of AF during the ablation procedure compared to the control group. However, this did not affect the recurrence rates of AF or atrial tachycardias within the 24-month follow-up period. Additionally, patients who underwent repeated ablation procedures exhibited a higher rate of maintaining sinus rhythm [68].

Extra-PV triggers are also found in the PW of the LA. Various atrial burst stimulation procedures, either with or without isoprenaline, have demonstrated ectopic atrial beats that start in the PW and induce AF [1]. The CAPLA study revealed that patients with PsAF who received PWI in addition to PVI did not show significant improvements after one year and at the long-term three-year follow-up, compared to those treated with PVI alone [1,60,69]. In another recent study, adding PWI to PVI with PFA showed no significant benefit over PVI alone at 1-year follow-up [63]. However, in those in whom the PW exhibited rapid activity, PWI was associated with significantly less arrhythmia recurrence [70]. Traditional endocardial ablation often fails to achieve durable transmural lesions in the PW. Epicardial mapping and ablation of the left atrial PW is gaining traction as a crucial strategy in managing PsAF, particularly within hybrid epicardial–endocardial approaches. The CONVERGE trial [71] demonstrated significantly improved arrhythmia-free survival at 1 year with a hybrid convergent procedure compared to endocardial ablation alone. Similarly, the CEASE-AF [72] trial showed that a staged hybrid approach incorporating epicardial PWI and left atrial appendage exclusion resulted in superior rhythm control in patients with PsAF compared to the endocardial-only approach, and this benefit persisted consistently over 3 years [73]. La Fazia et al. investigated the efficacy of different radiofrequency power settings (40 W vs. 50 W vs. 90 W) in achieving durable transmural lesions for PWI in patients with PsAF [74]. While endocardial mapping often showed apparent electrical silence in all groups, epicardial mapping revealed residual signals in most cases, except for the 50 W group, which achieved true transmural isolation in 83.3% of patients. The study highlighted the inferior PW as a key arrhythmogenic region often overlooked in endocardial ablation, emphasizing the need for targeted treatment in hybrid approaches and the importance of epicardial mapping for accurate lesion assessment [74].

The ablation of CFAE can be considered a potential target for PsAF. Several studies have investigated whether targeting specific subsets of CFAE may offer benefits. In the STAR AF II study, Verma et al. conducted a prospective randomized multicenter study that included 589 patients with PsAF, who underwent PVI alone, PVI with ablation of CFAEs, or PVI and additional linear ablation. Compared to patients receiving only PVI, patients receiving PVI and linear ablation or PVI and CFAEs experienced a considerably higher rate of acute cessation of AF. However, at the 18-month follow-up, there was no difference in the three groups’ rates of freedom from AF [75]. In a secondary analysis of the STAR AF II study, catheter ablation significantly reduced AF burden and improved quality of life, correlating with the percentage reduction in AF burden after the procedure [76]. Some meta-analyses showed that CFAE ablation did not improve outcomes in terms of AF-free survival [77,78,79]. The STAR AF II trial had a significant limitation: it did not adopt a personalized approach to treatment. All patients received standardized procedures, either PVI alone, PVI combined with CFAE ablation, or PVI along with linear ablation. This method did not consider the individual differences in atrial substrate, fibrosis, or electrophysiological characteristics. Such a “one-size-fits-all” strategy may have reduced the effectiveness of the additional ablation techniques, as more recent studies suggest that tailoring ablation based on patient-specific factors could lead to better outcomes. The reproducibility of CFAE ablation is inconsistent, and not all fractionated signals contribute equally to AF maintenance [80,81,82,83]. There is still no consensus on defining CFAE or which electrograms to ablate. Extensive CFAE ablation can create non-contiguous lesions, potentially causing slow conduction and increasing the risk of post-procedural atrial tachycardias [80,84]. This indicates that CFAEs may be passive areas influenced by wavebreak dynamics rather than active in maintaining AF [80]. While precise targeting of AF mechanisms remains unclear, further insights and improved mapping algorithms are needed to guide electrogram identification.

Other trials aimed to demonstrate that removing or modifying arrhythmogenic substrate can reduce the recurrence of atrial tachyarrhythmias. Non-transmural lesions resulting from endocardial ablation, along with various myocardial layers oriented differently, can act as substrates that induce arrhythmias [85]. KN Lee et al. showed that for redo procedures in patients with PsAF, combined epicardial and endocardial ablation was not superior to lone endocardial ablation regarding recurrence [85]. However, hybrid approaches with minimally invasive surgical epicardial ablation were associated with fewer recurrences in a randomized trial [71]. A recent meta-analysis found that surgical and catheter ablation have similar long-term success in preventing AF recurrence, with about 50% of patients remaining AF-free five years after treatment [86].

Alterations in the structure of Bachmann’s bundle (BB), such as a loss of the parallel alignment of muscle fibers, can promote re-entry circuits and contribute to the onset of AF [87,88]. An adjunctive BB epicardial ablation after endocardial catheter ablation, as a two-staged hybrid strategy, for patients with long-standing PsAF was shown to be a safe and highly effective approach, with 96.6% of patients remaining free from arrhythmia after 1 year [87]. A retrospective study found that adding BB modification to PVI using an endocardial approach in patients with PsAF resulted in 83.3% of patients experiencing no arrhythmia recurrences at 1 year [89].

Scar characterization is achieved through electro-anatomical mapping, which identifies low-voltage areas (LVAs), and late gadolinium CMR of the atrium [90,91]. A key limitation is how scar is defined, with low-voltage cut-offs ranging from 0.05 to 0.5 mV. The thin atrial wall makes accurate CMR scar threshold definitions challenging, potentially leading to discrepancies between regions identified by mapping and CMR [90,91]. Advancements in understanding AF pathophysiology indicate that structurally remodeled atrial areas with low bipolar mapping voltage may serve as substrates for reentry [92]. Additionally, the extent of LA fibrosis proved to be a strong predictor of AF recurrence following RFA [93]. The ERASE-AF (Low-Voltage Myocardium-Guided Ablation Trial of Persistent Atrial Fibrillation) study was a multicenter, randomized trial involving 324 patients with PsAF and demonstrated that PVI combined with personalized ablation of atrial LVA enhanced arrhythmia-free survival [92]. Other studies have reported different findings regarding the effectiveness of LVA ablation in PsAF (Table 1).

In the DECAAF II trial, CMR-guided fibrosis-targeted ablation with PVI did not significantly reduce atrial arrhythmia recurrence compared to PVI alone. Targeting additional fibrotic areas outside the PV ostia was also linked to higher ischemic stroke events [31]. The SUPPRESS-AF randomized trial showed that LVA ablation in addition to PVI did not significantly reduce 1-year AF or atrial tachycardia recurrence in patients with PsAF [94].

**Table 1:** The main studies that analyzed substrate ablation of atrial low-voltage areas.

Author (Year)	Study Design	No of Patients Included	Follow-Up Period	VGA Group Strategy (No of Patients)	Control Group Strategy (No of Patients)	Primary Outcome	Rhythm Control Outcome After the First Procedure (VGA vs. Control)
Wang et al. (2014) [95]	RCT	124	12 months	PVI + LVA (64)	PVI + stepwise (60)	All-atrial tachycardia recurrence	65.5% vs. 45% (*p* = 0.04)
Cutler et al. (2016) [96]	Retrospective	141	12 months	PVI + LVA (65)	PVI (66)	All-atrial tachycardia recurrence	80% vs. 57% (*p* = 0.005)
Yang et al. (2016) [97]	Retrospective	164	30 months	PVI + LVA (86)	PVI + stepwise (78)	All-atrial tachycardia recurrence	69.8% vs. 51.3% (*p* = 0.011)
Jadidi et al. (2016) [30]	Prospective	151	13 months	PVI + LVA (85)	PVI (66)	All-atrial tachycardia recurrence	69% vs. 47% (*p* < 0.001)
Yamaguchi et al. (2016) [98]	Retrospective	55	18 months	PVI + LVA (39)	PVI (16)	All-atrial tachycardia recurrence	72% vs. 79% (*p* = 0.4)
Yang et al. (2017) [99]	RCT	229	18 months	PVI + LVA (114)	PVI + stepwise (115)	All-atrial tachycardia recurrence	74% vs. 71.5% (*p* = 0.325)
Kumagai et al. (2019) [100]	RCT	54	24 months	PVI + BOXI + LVA (33)	PVI + BOXI (21)	All-atrial tachycardia recurrence	67% vs. 62% (*p* = 0.722)
Nery et al. (2020) [101]	Retrospective	145	18 months	PVI + LVA (95)	PVI (50)	All-atrial tachycardia recurrence	72% vs. 58% (*p* = 0.02)
Liu et al. (2021) [102]	Retrospective	136	18 months	PVI + LVA-guided linear ablation (97)	PVI + non-LVA guided linear ablation (39)	All-atrial tachycardia recurrence	83% vs. 62% (*p* = 0.043)
Hwang et al. (2021) [103]	RCT	50	12 months	PVI + LVA-CFAE (25)	PVI (25)	All-atrial tachycardia recurrence	60% vs. 40% (*p* = 0.329)
Suzuki et al. (2022) [104]	Retrospective	128	9.3 months	PVI + LVA (64)	PVI + linear (64)	AF or atrial flutter recurences	45% vs. 23% (*p* = 0.014)
Huo et al. (2022) [92]	RCT	324	12 months	PVI + LVA (161)	PVI (163)	All-atrial tachycardia recurrence	50% vs. 35% (*p* = 0.01)
Masuda et al. (2025) [94]	RCT	342	12 months	PVI + LVA (170)	PVI (171)	All-atrial tachycardia recurrence	61% vs. 50% (*p* = 0.127)

RCT—randomized controlled trial; VGA—voltage-guided ablation; PVI—pulmonary vein isolation; LVA—low-voltage ablation; AF—atrial fibrillation.

The limited effectiveness of fibrosis-guided ablation may result from technical challenges and the complex nature of AF. While fibrosis is linked to AF, its exact role in initiating or sustaining the condition is unclear [31]. Different types of fibrosis can vary in their arrhythmogenic potential, but current imaging cannot distinguish between them. Ablation of fibrotic tissue may not fully eliminate its arrhythmogenic properties, and fibrosis can progress beyond treated areas [31,94,105]. LVA ablation may miss other arrhythmogenic substrates, which can continue to sustain AF and contribute to treatment failure [94]. On the other hand, extensive ablation strategies, such as LVA ablation, may raise the risk of iatrogenic atrial tachycardias [94]. Unlike PVI, fibrosis ablation lacks standardization and clear procedural endpoints, leading to variable outcomes among operators [31].

The extensive LA ablation raises the risk of stiff LA physiology, which subsequently increases the AF recurrence rate [106,107,108]. Lee et al. found that stiff LA physiology after catheter ablation for AF occurred in 3.7% of cases, linked to low LA voltage and extra-PV ablation [106]. An observational study found LA calcification in 13.8% of AF ablation patients, often at the PV antrum, and that it is associated with multiple ablation procedures and increased estimated pulmonary pressure [109].

## 5. Other Energy Sources

In addition to RFA, other major energy sources used in AF ablation include cryoablation and PFA. In PsAF, atrial remodeling, fibrosis, and LA fat infiltration may impair radiofrequency delivery and adequate lesion formation; therefore, cryoablation and PFA may be more efficient. However, PsAF often necessitates personalized and substrate-based ablation techniques beyond PVI. Currently, one-shot techniques lack the flexibility to customize lesion sets. Therefore, while cryoablation and PFA are ideal for PAF, they may be less effective for PsAF until advancements allow for tailored substrate targeting (Table 2).

Both cryoablation and PFA have demonstrated efficacy and safety in the treatment of PsAF. Cryoballoon ablation offers shorter procedure times and a well-established safety profile, making it a viable option for many patients. PFA, being a newer technology, has shown promising results with potentially lower complication rates and is gaining traction as a treatment modality for PsAF. Additional randomized controlled trials are necessary to compare these two techniques and establish definitive treatment guidelines.

The STOP Persistent AF trial reported a 54.8% freedom from atrial arrhythmias at 12 months, with a low complication rate of 0.6% [110]. Additionally, the CRYO4PERSISTENT trial showed a 60.7% freedom from all atrial arrhythmias at 12 months, with a complication rate of 4% and no cases of phrenic nerve injury at discharge [111].

PFA is an emerging nonthermal ablation technique that has shown promising results in treating PsAF. This technique offers a major advancement in AF treatment through cardiomyocyte-selective ablation, safely targeting cardiac tissue while sparing surrounding structures. Reddy et al.’s studies emphasize the groundbreaking potential of PFA in treating AF. In 2020, they expanded the use of PFA to PsAF by combining PVI with ablation of the left atrial PW. This approach resulted in high lesion durability and a low rate of complications, further supporting the effectiveness of PFA in managing more complex AF cases [112]. A one-year follow-up study confirmed these outcomes, showing sustained PVI in most patients and a notable decrease in AF recurrence. These findings helped ease concerns about potential long-term clinical effects associated with PFA’s non-thermal ablation mechanism [113]. In patients with PAF, Reddy et al. showed that PFA was as effective and safe as thermal ablation at 12 months, with similar rates of arrhythmia recurrence, redo procedures, and serious adverse events [114].

The EU-PORIA registry provided real-world results from seven high-volume AF ablation centers in Europe, showing high procedural success and a favorable complication profile, thus reinforcing the reproducibility of PFA in clinical practice [115]. The PULSED AF pivotal trial that included 150 patients with PsAF reported a 55.1% freedom from atrial arrhythmias at 12 months, with a low complication rate of 0.7%, with no cases of esophageal injury, phrenic nerve injury, PV stenosis, or atrio-esophageal fistula [116]. The MANIFEST-PF study, involving 1758 real-world patients across multiple European centers, confirmed 99.9% PVI success and zero esophageal or permanent phrenic injuries, validating PFA’s tissue specificity. These safety advantages make PFA particularly promising for PsAF, where more extensive lesion sets (e.g., PW or mitral isthmus) are often required [117]. The MANIFEST-PF registry’s one-year outcomes showed a 78.1% freedom from atrial arrhythmias and a 1.9% incidence of acute major adverse events, with greater clinical effectiveness in PAF than in PsAF [118]. Furthermore, a prospective study involving 32 patients with PsAF demonstrated a 65.6% freedom from atrial arrhythmias at 1 year, with high acute success rates for PVI and left atrial PWI [119]. In the PULSE-EU study, a novel single-shot spherical array PFA system achieved 80% freedom from atrial arrhythmia at 1 year in PsAF patients, with 100% acute PVI after an average of 1.2 applications per pulmonary vein [120]. Della Rocca et al. evaluated a tailored PFA protocol using a multi-electrode catheter to systematically target PVs, the PW, and low-voltage regions in patients with PsAF and long-standing PsAF. This approach resulted in high rates of AF termination (95.8%) and excellent arrhythmia-free survival at 1 year (89.2%) with low complication rates, demonstrating the potential benefit of personalized substrate modification [121]. The SPHERE Per-AF trial was a randomized trial comparing a dual-energy lattice-tip catheter system (combining PFA and radiofrequency) to conventional RFA. The novel system achieved non-inferior effectiveness (73.8% vs. 65.8%, *p*  <  0.0001) and safety, with shorter procedure times and comparable complication rates [122]. These findings support targeted substrate modification and advanced ablation technologies to improve PsAF outcomes. Therefore, PFA may enable a significant shift in treating PsAF by allowing for safer expanded lesion sets, minimizing complication risks, and enhancing procedural efficiency; however, long-term outcomes still need to be assessed in ongoing trials.

## 6. Can Machine Learning and Artificial Intelligence Improve Patient Selection and Downstream Clinical Outcomes?

Machine learning (ML) and artificial intelligence (AI) have significant potential to enhance patient selection and clinical outcomes in managing PsAF, especially in the context of catheter ablation.

### 6.1. Patient Selection

Traditionally, selecting appropriate candidates for ablation has relied on clinical parameters and physician experience, which can be limited in predicting long-term success. AI offers the ability to analyze large, complex datasets—including ECGs, imaging (such as CMR and voltage mapping), wearable device outputs, and clinical history—to identify patterns that predict which patients are most likely to benefit from ablation [123,124]. Furthermore, AI can assist in personalizing ablation strategies by detecting arrhythmogenic substrates like LVAs, rotors, or extra-PV triggers, thus optimizing lesion placement [125]. Predictive models developed through ML can also estimate the risk of recurrence, procedural complications, or post-ablation atrial tachycardias, enabling more informed decision-making and follow-up planning [123,126]. A retrospective study (321 patients; ~60% PsAF) built an ML model combining LA CT imaging features with clinical data to predict 1-year ablation outcomes. The authors conclude that this deep-learning approach can improve the identification of ablation “responders,” aiding personalized patient selection for PsAF ablation [127].

Park et al. found that a larger gap between AI-predicted ECG age and chronological age reliably predicts AF recurrence after catheter ablation. This association held across diverse cohorts and remained consistent regardless of patient age or LA size, suggesting the ECG age gap could serve as a simple, interpretable risk marker for post-ablation recurrence [128]. Deep neural networks that incorporate intracardiac signals, 12-lead ECG data, and clinical variables can enhance the accuracy of predicting catheter ablation outcomes. Tang et al. reported that deep convolutional neural networks trained on electrogram or ECG data have shown superior performance in predicting catheter ablation outcomes compared to conventional clinical scores, with prediction accuracy further enhanced by combining electrogram, ECG, and clinical features [129].

Another recent study of 561 AF ablation patients (67% nonparoxysmal) using ML found a poor correlation between acute procedural success and long-term outcomes [130]. While 49.6% achieved acute AF termination during ablation, 1-year freedom from AF was around 69.5% [130]. Feature importance analysis revealed that clinical and lifestyle factors mainly influenced long-term success, while acute success was linked to electrophysiological features [130]. This indicates that distinct factors govern acute and long-term outcomes, highlighting the need for new strategies to enhance long-term predictions beyond immediate procedural success.

### 6.2. Substrate Mapping

ML-enhanced electroanatomic mapping identifies critical arrhythmogenic substrates in PsAF by analyzing intracardiac electrograms to find LVAs and fibrosis. These tools use pattern recognition and clustering algorithms to guide targeted ablation beyond standard PVI, enhancing precision and reducing inter-operator variability.

The VOLTA VX1 software is an AI-driven solution that uses ML to identify potential arrhythmogenic substrates from electrogram data and offers real-time insights for physicians into areas with possible dispersion (DISPERS) characteristics. Bahlke et al. showed that DISPERS-guided ablation using the Volta VX1 software in addition to PVI in long-standing PsAF ablation resulted in high 12-month success rates regarding AF elimination [131].

A post hoc sub-analysis of the EARNEST-PVI trial demonstrated that uplift modeling via ML can effectively identify a subgroup of patients with PsAF who are most likely to benefit from more extensive ablation strategies, including linear ablation and/or CFAE ablation [132]. Forward-solution AI ECG mapping has been shown to decrease the time to first ablation, shorten overall procedure duration, and reduce fluoroscopy exposure, while maintaining comparable arrhythmia-free survival rates and complication rates [133].

The TAILORED-AF trial is the first large-scale, randomized controlled trial demonstrating that AI-guided ablation targeting spatiotemporal electrogram dispersion (DED) in addition to PVI offers superior outcomes compared to PVI alone in PsAF [134]. The tailored strategy was particularly effective in patients with more advanced disease, without compromising safety, suggesting a promising step toward a personalized ablation approach. Seitz et al. demonstrated that an AI-based software solution can effectively standardize expert-based electrogram analysis, resulting in consistent electrogram-based ablation outcomes [135]. In another recent study, Seitz et al. showed that the distribution and extent of DED in the atria display a distinct, nonrandom pattern unique to each patient with PsAF, and AI-DED could act as a recognizable marker of the substrate [136]. AI-guided dispersion mapping can reveal atrial remodeling beyond traditional voltage mapping, enhancing AF substrate characterization. In a study of 85 AF patients (paroxysmal and persistent) undergoing high-density mapping, an AI algorithm identified spatiotemporal dispersion regions in 92% of cases [137]. Notably, 42% had dispersion areas extending beyond low-voltage zones, and the total dispersion correlated with atrial remodeling markers. PsAF patients had more dispersion sites and dispersion patterns that remained stable before and after cardioversion. At follow-up, 60% of persistent vs. 33% of paroxysmal patients experienced arrhythmia recurrence despite dispersion-guided ablation [137].

The FLOW-AF study tested Ablacon’s ML algorithm for patients with PsAF who had previous ablations [138]. The results after 12 months showed that 68% of patients with source-targeted ablation remained arrhythmia-free, compared to just 17% in the PVI-only group [138]. This indicates that those with identifiable extra-PV drivers significantly benefit from substrate-guided ablation, with real-time visualization confirming the elimination of sources.

### 6.3. Real-Time Procedural Guidance

AI systems improve ablation by monitoring catheter contact and optimizing energy delivery while assessing lesion quality in real-time.

Robotic magnetic navigation (RMN)-guided PVI offers high AF-free rates in PAF, comparable to manual RFA [139]. Li et al. and Luo et al. demonstrated that in 200 patients with PsAF who underwent their first AF ablation, cryoablation is comparable to RMN-guided ablation in terms of mid-term and long-term freedom from atrial tachycardias [140,141].

Preliminary research is exploring the potential of AI to recommend optimal ablation lesion sets tailored to individual patients. A study by Ogbomo-Harmitt et al. utilized deep learning techniques on patient-specific atrial models derived from LGE-CMR scans to compare three different ablation strategies: PVI alone, PVI combined with fibrosis-guided lines, and PVI alongside rotor-guided ablation. Their convolutional neural network predicted which strategy was most likely to terminate AF based on the characteristics of the atrial substrate, revealing that the fibrosis-guided strategy had the highest success rate [142]. This study highlights the potential of AI-driven planning tools to analyze atrial imaging, patient factors, and EGM for personalized ablation strategies.

Ensuring PVs are truly isolated can be challenging in real time due to far-field signals and operator subjectivity. The PVISION multicenter trial validated an ML algorithm that assessed PVI status from high-fidelity intracardiac signals and accurately identified the moment of isolation in about 87% of cases [143]. For instance, the VX1 dispersion mapping software used in TAILORED-AF provides real-time AI analysis of electrograms, instantly highlighting regions of complex fractionation/spatiotemporal dispersion while the patient is in AF [134].

Although still in the experimental stage, these tools hold the potential to enhance procedural efficacy and offer real-time decision support.

## 7. Conclusions

PVI is essential but often insufficient in PsAF, prompting the use of a personalized, substrate-based strategy to address the complex arrhythmogenic substrate. Recent advancements in high-resolution mapping technologies have significantly improved the identification and targeting of arrhythmogenic areas, leading to better procedural results. The empiric ablation approaches have shown promising results and may provide incremental benefit when added to PVI until further AF mechanistic understanding is achieved.

Energy sources in PsAF ablation are evolving, with RFA remaining the gold standard and PFA showing potential as a tissue-selective alternative. As technology advances, the selection of energy will be tailored to each patient’s anatomy, substrate, and procedural goals. Though still evolving, AI and ML are expected to play important roles in PsAF management.

## Figures and Tables

**Table 2:** Comparison of techniques used for the ablation of patients with persistent atrial fibrillation.

	Radiofrequency Ablation (RFA)	Cryoablation	Pulsed Field Ablation
Energy type	Thermal (heating)	Thermal (freezing)	Non-thermal (electroporation)
Mechanism of action	Resistive heating → necrosis	Freezing → cellular injury	Electroporation → myocardial-specific membrane disruption
Delivery	Point-by-point (catheter)	Single-shot (balloon)	Single-shot (balloon or multi-electrode array) and point-by-point (catheter)
Optimal use in PsAF	PVI + substrate modification	PVI, PWI	PVI, PWI, mitral isthmus
Advantages	○ The standard approach for complex substrate ablation	○ Uniform lesion formation with less risk of PV stenosis. ○ Shorter procedure time	○ Tissue selectivity ○ Rapid lesion creation ○ Minimal collateral damage ○ Very low risk of esophageal injury ○ Shortest procedure time
Limitations	○ Highly operator-dependent ○ Risk of collateral damage ○ Longest procedure time	○ Limited ability to modify extra-PVs substrate—may require adjunctive RFA ○ Moderate risk of esophageal injury	○ Still under clinical investigation for widespread use beyond PVI. ○ Long-term outcomes in PsAF are still being evaluated

RFA—radiofrequency ablation; PVI—pulmonary vein isolation; PVs—pulmonary veins; PsAF—persistent atrial fibrillation; PWI—posterior wall isolation.
